# Evaluation of myocardial work in patients with hypertrophic cardiomyopathy and hypertensive left ventricular hypertrophy based on non-invasive pressure-strain loops

**DOI:** 10.3389/fcvm.2022.767875

**Published:** 2022-07-26

**Authors:** Qingqing Zhao, Cunying Cui, Yanan Li, Yuanyuan Liu, Danqing Huang, Ying Wang, Yanbin Hu, Ruijie Liu, Huizhen Zhu, Lin Liu

**Affiliations:** Department of Ultrasound, Henan Key Laboratory of Cardiovascular Ultrasound Clinical and Basic Research, Fuwai Central China Cardiovascular Hospital, Central China Fuwai Hospital of Zhengzhou University, Henan Provincial People's Hospital, People's Hospital of Zhengzhou University, Zhengzhou, China

**Keywords:** hypertrophic cardiomyopathy, hypertension, echocardiography, pressure-strain loops, myocardial work, left ventricular

## Abstract

**Background:**

The capacity to distinguish hypertrophic cardiomyopathy (HCM) from hypertensive left ventricular hypertrophy (H-LVH) based on morphological features obtained by conventional echocardiography is limited. We investigated the global myocardial work of the left ventricle in two types of hypertrophies using the non-invasive myocardial work index (NMWI).

**Methods:**

Conventional echocardiography was performed on 107 subjects with preserved left ventricular ejection fraction (LVEF ≥ 50%), who comprised patients with HCM (*n* = 40), H-LVH (*n* = 35), and healthy people with normal blood pressure and left ventricular structure (*n* = 32). Except for the conventional echocardiographic parameters, the left ventricular myocardial work parameters based on pressure-strain loops, including global myocardial work index (GWI), global constructive work (GCW), global wasted work (GWW), and global work efficiency (GWE), were evaluated in three groups. Multivariate discriminant analysis and receiver operating characteristic (ROC) curve were used to evaluate the incremental value of NMWI for distinguishing HCM from H-LVH.

**Results:**

Compared to the control group, GWI and GCW were significantly lower in HCM patients (*P* < 0.05), whereas GWI was significantly higher in H-LVH patients. GWW was higher and GWE was significantly decreased in both HCM and H-LVH patients than in the control group (*P* < 0.05). Multivariate discriminant analysis and ROC curve revealed that the inter-ventricular septum thickness (IVST)/left ventricular posterior wall thickness (LVPWT) and GCW were each able to distinguish HCM from H-LVH. The combination of IVST/LVPWT and GCW discriminated HCM and H-LVH with a higher predictive accuracy of 94.7%.

**Conclusion:**

NMWI may provide additional information in evaluating the myocardial function in patients with HCM and H-LVH. Myocardial work combined with conventional echocardiography could improve the clinical diagnostic accuracy of distinguishing HCM and H-LVH.

## Introduction

Hypertrophic cardiomyopathy (HCM) is an autosomal dominant hereditary disease characterized mainly by ventricular wall hypertrophy with an unknown cause (cardiac afterload should be excluded), and it is one of the main causes of sudden cardiac death (1). Hypertension heart disease can cause structural and functional remodeling of the myocardium, leading to cardiac systolic and diastolic dysfunction and eventually to heart failure (2). Left ventricular hypertrophy (LVH) is the common pathway of cardiac damage in primary hypertension due to long-term increased afterload, which is associated with clinical prognosis. Both HCM and hypertension LVH (H-LVH) patients exhibit left ventricular wall thickening, and other similar characteristics in conventional echocardiography, but their pathogenesis, treatment, and prognosis are different (3).

Previous studies have shown that global longitudinal strain (GLS) is a sensitive index for evaluating the left ventricular systolic function and it has potential clinical value for differentiating various types of LVH diseases (4, 5). However, the strain is load-dependent and an increase in the afterload will lead to a decrease in GLS, thereby affecting the accuracy of the research results (6). Non-invasive myocardial work index (NMWI) is a new method for myocardial function assessment based on the two-dimensional speckle tracking technique, as well as considering myocardial deformation and left ventricular pressure, and reduces the effect of afterload on strain (7).

We hypothesized that NMWI might provide incremental value along with conventional echocardiographic parameters for discrimination between HCM and H-LVH. The aim of the present study was to evaluate the difference in myocardial work between patients with HCM and H-LVH and determine whether NMWI is able to provide more detailed information on the systolic function of the left ventricle and possible application for differential diagnosis.

## Materials and methods

### Study population

Seventy-five patients with left ventricular hypertrophy from the Fuwai Central China Cardiovascular Hospital from March 2019 to April 2020 were selected. Patients were diagnosed with non-obstructive HCM (*n* = 40, 70% men, age 46.3 ± 10.7 years) or H-LVH (*n* = 35, 68% men, age 48.1 ± 14.0 years). All drugs were discontinued at least 3 days before the patients' evaluation. In addition, age- and gender-matched healthy individuals with normal electrocardiography, echocardiography, and physical examination and without a history of cardiovascular disease were selected as the control group (*n* = 32, 65% men, age 45.3 ± 6.7 years).

The diagnosis of HCM was established according to current guidelines (1), i.e., each patient had one or more regions with a maximal wall thickness of left ventricle ≥ 15 mm (with family history ≥ 13 mm) that was not explained solely by loading conditions. Non-obstructive HCM was defined as the left ventricular outflow tract gradient <30 mm Hg at rest or excited states, excluding myocardial hypertrophy caused by other cardiac or systemic diseases.

The diagnosis of H-LVH was based on the ESC/ESH arterial hypertension management guidelines in 2018 (8), i.e., systolic blood pressure ≥ 140 mm Hg and/or diastolic blood pressure ≥ 90 mm Hg (1 mm Hg = 0.133 kPa) before medication or current therapy with antihypertensive drugs. Left ventricular mass index (LVMI) > 115 g/m^2^ (male)/95 g/m^2^ (female).

All subjects whose sinus rhythm, left ventricular ejection fraction (LVEF) ≥ 50%, and coronary angiography or coronary computed tomographic angiography showed that the degree of stenosis in the three coronary arteries was <50% were included. Patients with secondary hypertension, New York Heart Association (NYHA) grade III-IV, diabetes, structural heart disease, other types of cardiomyopathies (dilated cardiomyopathy, myocardial amyloidosis, etc.), and severe systemic diseases were excluded. This study was approved by the Ethics Committee of Fuwai Central China Cardiovascular Hospital, and all subjects gave their signed informed consent.

### Conventional echocardiography

A GE Vivid E95 (GE Vingmed Ultrasound AS, Horten, Norway) color Doppler ultrasound diagnostic instrument equipped with an M5 Sc-D probe 1.4–4.6 MHz transducer was used. The limb lead electrocardiogram was connected synchronously and patients were in the left lateral decubitus position. The left atrial diameter (LAD), left ventricular end-systolic diameter (LVSd), left ventricular end-diastolic diameter (LVDd), inter-ventricular septum thickness (IVST), and left ventricular posterior wall thickness (LVPWT) were measured routinely. Left ventricular end diastolic volume (LVEDV) and LVEF were measured using the biplane Simpson's rule. LVMI and relative wall thickness (RWT) were calculated as follows: LVMI = left ventricular mass (LVM)/BSA; LVM = 0.8 × {1.04[(LVDd + IVST + LVPWT)3 – LVSd3]} + 0.6; RWT = (IVST + LVPWT)/LVDd. The flow spectrum was collected for the aortic valve and mitral valve, and peak early and late diastole velocity of the mitral valve and the ratio (E/A) were measured.

### Myocardial work analysis

Two-dimensional grayscale images from the apical four-chamber, two-chamber, and three-chamber views were acquired continuously for at least three cardiac cycles at a frame rate of 61.2 ± 7.1 frames/s on average to enable GLS and myocardial work analysis by STE. And the images were collected and stored on a hard disk in original format for offline analysis.

**Figure 1:**
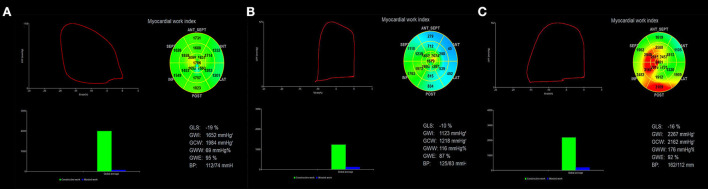
Assessment of left ventricular myocardial work using NMWI. The red curve in the upper left of the figure shows the relationship between pressure and strain in the left ventricle, and its area represents GWI. The histogram at the lower left shows GCW and GWW values. The figure in the upper right shows a bullseye diagram based on the work index for 17 segments of the left ventricle. The results obtained based on the global myocardial parameters are shown at the bottom right. **(A)** Myocardial work results obtained in the control group show that the myocardial work distribution in each segment was consistent (uniform green). **(B)** HCM patients had a smaller left ventricular pressure-strain loop, and the blue area represents the decreased myocardial work. **(C)** The left ventricular pressure-strain loop increased in patients with H-LVH and the red color indicates the enhanced myocardial work. NMWI, non-invasive myocardial work index; GWI, global work index; GCW, global constructive work; GWW, global wasted work; GWE, global work efficiency.

Myocardial work was estimated using a commercially available software package (Echopac Version 203, GE Vingmed Ultrasound), which was constructed from a surrogate of the left ventricular pressure curve combined with GLS acquired with speckle tracking echocardiography, as proposed by Russell et al. (9, 10). The dynamic images of the apical three-chamber, four-chamber, and two-chamber views were then selected. The software automatically outlined the endocardium and LV wall. The tracking effect was carefully and dynamically observed. If the tracking was not satisfactory, tracking points were adjusted manually to outline the entire myocardial thickness. LV strain data were obtained automatically. Blood pressure was measured from the brachial cuff by a sphygmomanometer, assuming that the peak systolic pressure was equal to the peak arterial pressure. The software then constructed a non-invasive pressure curve adjusted according to the duration of isovolumic and ejection phases defined by the timing of aortic and mitral valve opening and closing events on echocardiography. The LV strain and pressure data were then synchronized through alignment of valvular timing events and systolic blood pressure. The left ventricular pressure-strain loop (PSL) and the following myocardial work parameters were provided automatically by the software, as shown in Figure 1.

The PSL area represented GWI and indicated the work done from mitral valve closure to mitral valve opening. The work done by the myocardial cell of shortening during systole and elongating during isovolumic diastole was helpful for left ventricular contraction and relaxation represented GCW. The work done by the myocardial cells of elongating during systole and shortening during isovolumic diastolic myocardium was not conducive to left ventricular pumping represented GWW, and GWE was the ratio of GCW to the sum of GCW and GWW (11).

The average time needed to analyze the myocardial work parameters on each patient was approximately 197 ± 48 s.

### Reproducibility of NMWI parameters

Twenty subjects were randomly selected and analyzed at different times by an observer blinded to the results of previous measurements for the intra-observer variability test. The same images were analyzed by other physicians blinded to the values obtained by the first observer for the inter-observer variability test.

### Statistical analysis

Continuous variables are expressed as mean and standard deviation when normally distributed. One-way analysis of variance (ANOVA) was used to conduct comparisons among three groups, followed by Fisher's least significant difference (LSD)*-t-*test for pairwise comparison. When not normally distributed, expressed as median (interquartile range [IQR]) and the rank sum test was used for comparisons. Categorical data were expressed in terms of frequencies and percentages and compared by the χ^2^-test.

The parameters for distinguishing HCM and H-LVH were screened by univariate analysis. Covariables examined included LAD, LVEF, IVST/LVPWT, LVDd, LVMI, E/A, GWI, GCW, GWW, and GWE. The parameters where *P* < 0.05 were entered into the multivariate stepwise discriminant analysis. The discriminant score (*Z*) and discriminant probability were calculated based on a discriminant function test. Optimal cutoff values, sensitivity, and specificity of parameters for differentiation between HCM and H-LVH were determined by the receiver operating characteristic (ROC) curve. Pearson's correlation analysis was adopted to assess the correlation between variables.

The Bland-Altman analysis was used for the repeatability test of MNWI parameters. SPSS 23.0 (SPSS Inc., IBM, Chicago, IL, USA) software was adopted for statistical analysis. *P* < 0.05 were considered to indicate statistical significance.

## Results

### General characteristics

Seventeen percent (7/40) of HCM patients had a history of or coexisting hypertension. The general clinical data of the study subjects are summarized in Table 1. The systolic blood pressure and diastolic blood pressure were significantly higher in H-LVH patients than in HCM and control groups (*P* < 0.05). There were no statistical differences among the three groups in age, sex, BSA, and heart rate (*P* > 0.05).

**Table 1:** General characteristics for study groups.

	**Control (*****n*** = **32)**	**HCM (*****n*** = **40)**	**LVH (*****n*** = **35)**	***P***
Age (y)	45.3 ± 6.7	46.3 ± 10.7	48.1 ± 14.0	0.56
Men, *n* (%)	21 (65)	28 (70)	24 (68)	0.31
BSA (m^2^)	1.79 ± 0.15	1.85 ± 0.17	1.81 ± 0.18	0.27
SP (mm Hg)	120.15 ± 6.04	124.07 ± 10.57	152.2 ± 18.97^*#^	<0.001
DP (mm Hg)	79.78 ± 5.36	83.80 ± 9.8	101.7 ± 15.7^*#^	<0.001
HR (bpm)	65.65 ± 5.94	64.70 ± 7.32	65.45 ± 4.51	0.77
Hypertention, *n* (%)	0 (0)	7 (17)	35 (100%)	<0.001
hyperlipidemia, *n* (%)	0 (0)	9 (22)	21 (60)	<0.001
Medications, *n* (%)
ACEI/ARB	-	8 (20)	21 (60)	-
Beta blocker	-	12 (30)	19 (54)	-
calcium channel blocker	-	11 (27)	18 (51)	-
Statin	-	9 (22)	25 (71)	-

BSA, body surface area; SP, systolic pressure; DP, diastolic pressure; HR, heart rate; ACEI, angiotensin converting enzyme inhibitor; ARB, angiotensin II receptor blocker. Data are expressed as mean ± SD. *P < 0.05 vs. control;

^#^P < 0.05 vs. HCM.

### Conventional echocardiography analysis

Table 2 compares the conventional echocardiography parameters among the three groups. Compared to the control group, significantly increased IVST, LVPWT, RWT, LAD, and LVMI, and markedly decreased E and E/A were observed in patients with HCM and H-LVH (*P* < 0.05). Furthermore, IVST, IVST/LVPWT, RWT, and LVMI were higher in HCM patients than in H-LVH patients (*P* < 0.05).

**Table 2:** Comparison of echocardiographic parameters for study groups.

**Parameter**	**Control**	**HCM**	**H-LVH**	***P***
IVST (mm)	9.47, 0.77	18.30, 4.15*	13.10, 1.35*^#^	<0.001
LVPWT (mm)	8.66, 0.65	12.14, 3.29*	11.54, 0.83*	<0.001
IVST/LVPWT	1.09, 0.05	1.60, 0.57*	1.14, 0.13^#^	<0.001
RWT	0.38, 0.04	0.64, 0.12*	0.49, 0.04*^#^	<0.001
LAD (mm)	30.68, 2.20	38.80, 3.83*	37.57, 3.64*	<0.001
LVMI (g/m^2^)	88.61, 17.23	155.94, 50.63*	129.07, 13.66*^#^	<0.001
LVDd (mm)	47.00, 3.21	46.00, 3.68	48.37, 4.10	0.11
LVSd (mm)	31.56, 3.21	30.65, 3.45	32.34, 3.36	0.10
LVEDV (ml)	102.93, 17.22	102.60, 19.62	106.77, 14.32	0.33
LVEF (%)	60.1, 6.19	62.31, 4.69	60.8, 5.59	0.25
E (m/s)	0.86, 0.15	0.66, 0.19*	0.71, 0.22*	<0.001
A (m/s)	0.67, 0.16	0.69, 0.27	0.80, 0.27	0.06
E/A	1.32, 0.26	1.07, 0.41*	0.94, 0.32*	<0.001

IVST, interventricular septal thickness; LVPWT, left ventricular posterior wall thickness; RWT, related wall thickness; LAD, left atrial diameter; LVMI, left ventricular mass index; LVDd, left ventricular end-diastolic diameter; LVSd, left ventricular end-systolic diameter; LVEDV, left ventricular end diastolic volume; LVEF, left ventricular ejection fraction; E, early diastolic velocity of mitral flow; A, late diastolic velocity mitral valve; Data are expressed as mean ± SD.

^*^P < 0.05 vs. control;

^#^P < 0.05 vs. HCM.

### GLS and myocardial work parameters analysis

**Figure 2:**
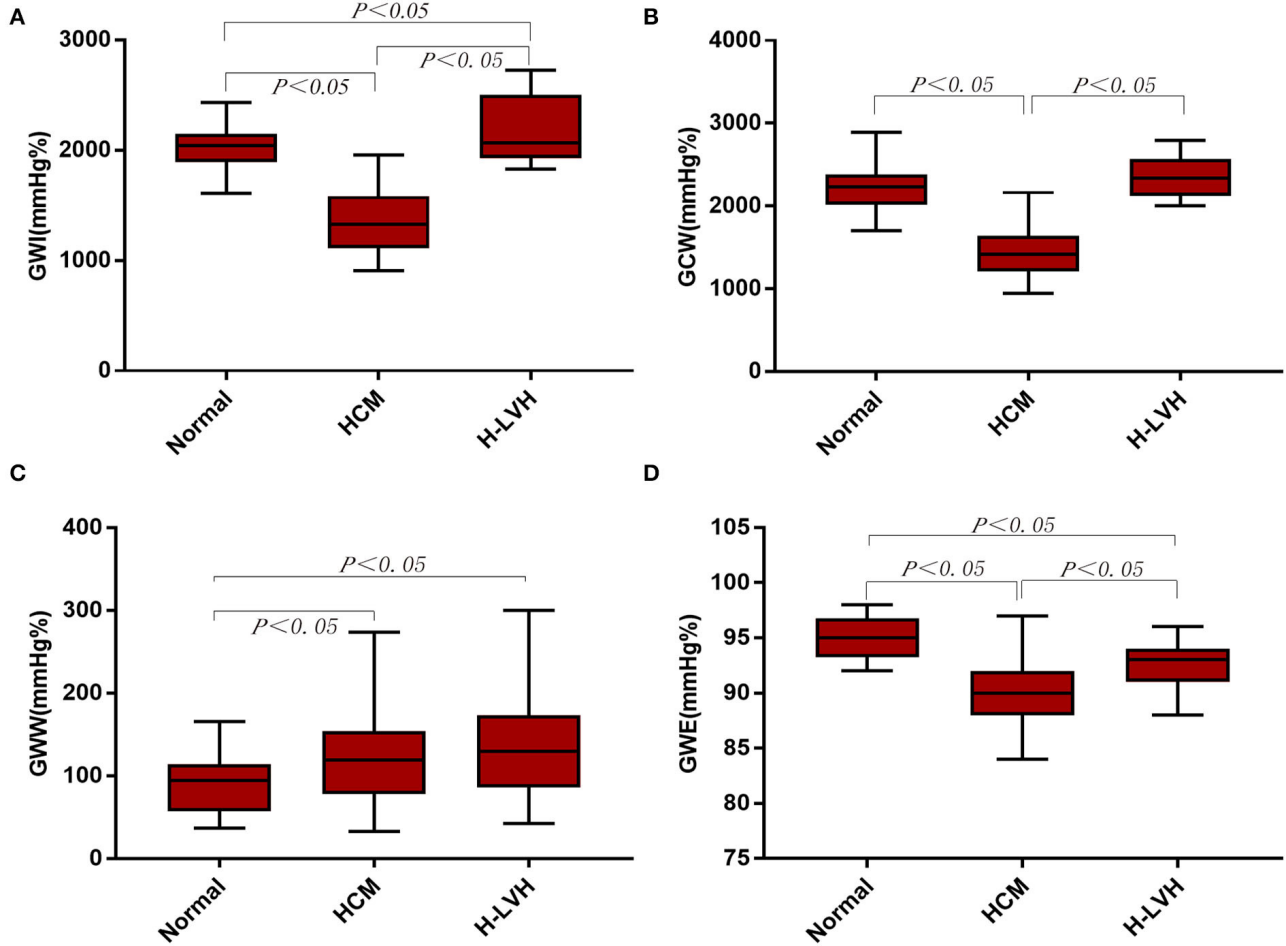
Left ventricular myocardial work in the control, HCM, and H-LVH groups. **(A)** GWI (global work index); **(B)** GCW (global constructive work); **(C)** GWW (global wasted work); **(D)** GWE (global work efficiency).

Compared to the control group, GWI and GCW were significantly lower in patients with HCM, and GWI was significantly higher in H-LVH patients (*P* < 0.05). Significantly decreased absolute values of GLS and GWE and markedly increased GWW was observed in patients with HCM and H-LVH (*P* < 0.05). The absolute value of GLS, GWI, GCW, and GWE were significantly decreased in patients with HCM than those in H-LVH patients (*P* < 0.05; Table 3 and Figure 2).

**Table 3:** Comparison of GLS and myocardial work parameters for study groups.

	**Control**	**HCM**	**H-LVH**	***P***
GLS (%)	−20.06, 1.88	−13.65, 2.76*	−16.74, 1.72*^#^	<0.001
GWI (mm Hg%)	2029.40, 214.04	1368.97, 283.79*	2210.77, 296.64*^#^	<0.001
GCW (mm Hg%)	2223.18, 265.05	1457.82, 315.10*	2350.77, 245.55^#^	<0.001
GWW (mm Hg%)	93.53, 39.58	124.37, 46.47*	137.1, 59.58*	<0.001
GWE (%)	95.06, 1.70	90.47, 3.12*	92.65, 2.10*^#^	<0.001

GLS, global longitudinal strain; GWI, global work index; GCW, global constructive work; GWW, global wasted work; GWE, global work efficiency; Data are expressed as mean ± SD.

^*^P < 0.05 vs. control;

^#^P < 0.05 vs. HCM.

### Multivariate discriminant analysis and ROC curve for distinguishing HCM from H-LVH

**Figure 3:**
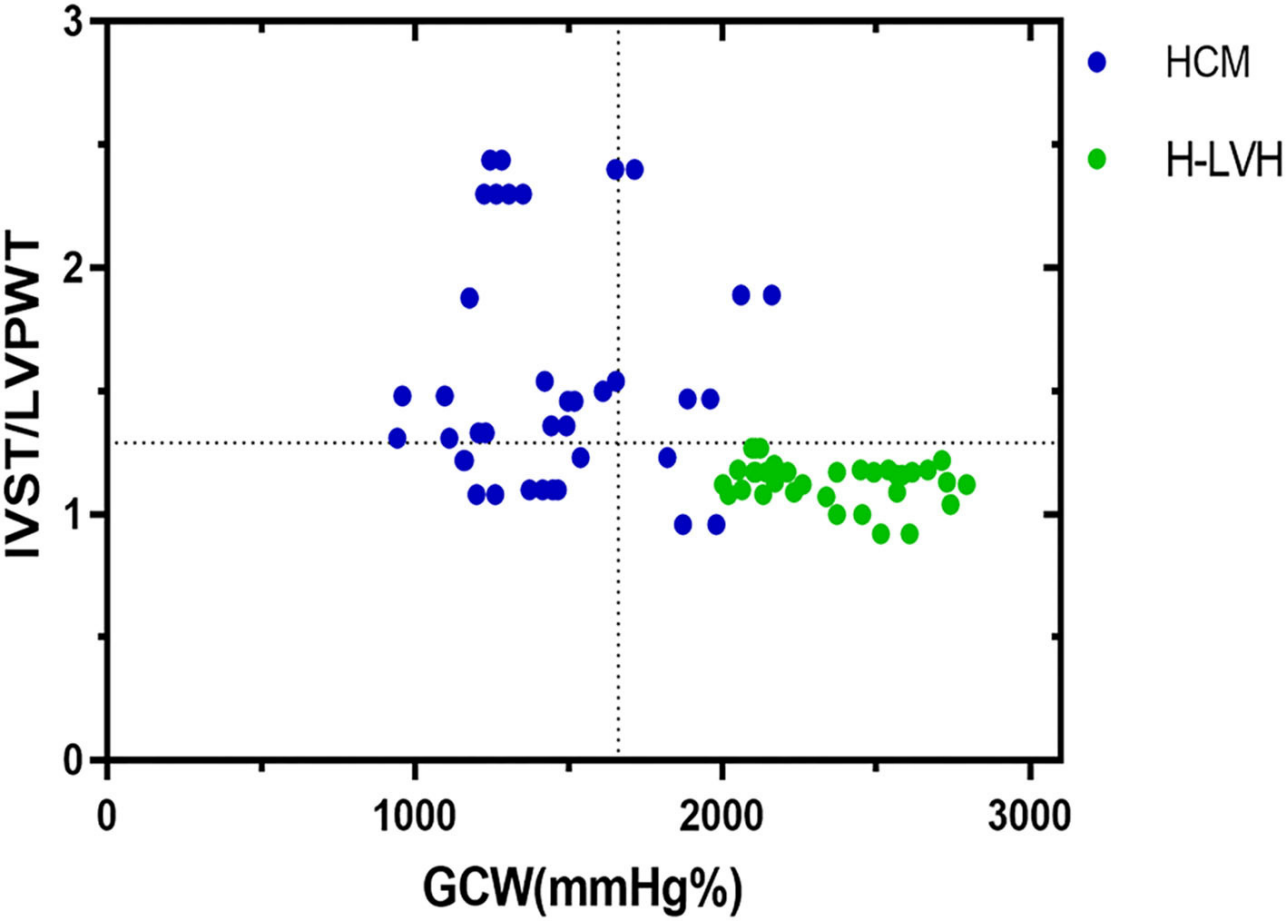
Relation between IVST/LVPWT ratio and GCW in patients with HCM and H-LVH. Optimal cutoff values for discrimination between the two groups of patients are indicated (dotted lines).

The parameters (IVST/LVPWT, LVMI, GWI, GCW, and GWE) that *P* < 0.05 according to univariate analysis were entered into multivariate stepwise discriminant analysis, which showed that IVST/LVPWT and GCW were independent predictors for distinguishing HCM from H-LVH. ROC curve analysis revealed that the cut-off values for IVST/LVPWT and GCW in the diagnosis of HCM were 1.29 and 1,662 mm Hg%, respectively, with sensitivity of 68.2 and 75.0%, specificity of 95.5 and 98.5%, and accuracy of 81.3 and 90.2%, respectively. According to the discriminant analysis results, the following discriminant function formula was obtained, and the discriminant score (*Z*) and discriminant boundary value were determined (Table 4 and Figure 3).


$$\begin{array}{l} Z=-4.979-0.935\times \left(IVST/LVPWT\right)+0.003\;\times \;GCW \end{array}$$

HCM was diagnosed when Z < −0.542, and H-LVH was diagnosed when Z > −0.542. In our study, the predictive accuracy of the discriminant formula based on IVST/LVPWT and GCW for diagnosing HCM and H-LVH was 94.7%, and interactive validation showed that the sensitivity of the discriminant function was 90.0% and the specificity was 100.0%.

**Table 4:** Multivariate discriminant analysis and ROC curve analysis for distinguishing HCM from H-LVH.

**Variable**	**Discriminant coefficient**	**AUC (95%CI)**	**Cut-off values**	**Sensitivity (%)**	**Specificity (%)**	**Predictive accuracy (%)**
IVST/LVPWT	−0.935	0.80 (0.73–0.93)	1.29	68.2	95.5	81.3
GCW (mm Hg%)	0.003	0.94 (0.89–0.98)	1662	75.0	98.5	90.1

IVST, interventricular septal thickness; LVPWT, left ventricular posterior wall thickness; GCW, global constructive work.

### Correlations between global myocardial work parameters and GLS/LVEF

GWI, GCW, and GWE were positively correlated with the absolute value of GLS (*r* = 0.76, 0.79, 0.67; *P* < 0.001), whereas GWW was negatively correlated with the absolute value of GLS (*r* = −0.59, *P* = 0.00). GWI, GCW, and GWE were positively correlated with LVEF (*r* = 0.51, 0.58, 0.39; *P* = 0.00), whereas GWW was negatively correlated with LVEF (*r* = −0.45, *P* = 0.00; Table 5).

**Table 5:** Relationship between myocardial work parameters and GLS/LVEF.

**Parameter**	**Absolute value of GLS(%)**		**LVEF(%)**	
	***R***	***P***	***r***	***P***
GWI (mm Hg%)	0.76	<0.001	0.51	<0.001
GCW (mm Hg%)	0.79	<0.001	0.58	<0.001
GWW (mm Hg%)	−0.59	<0.001	−0.45	<0.001
GWE (%)	0.67	<0.001	0.39	<0.001

GWI, global work index; GCW, global constructive work; GWW, global wasted work; GWE, global work efficiency; GLS, global longitudinal strain; LVEF, left ventricular ejection fraction.

### Intra-observer variability and inter-observer variability

**Figure 4:**
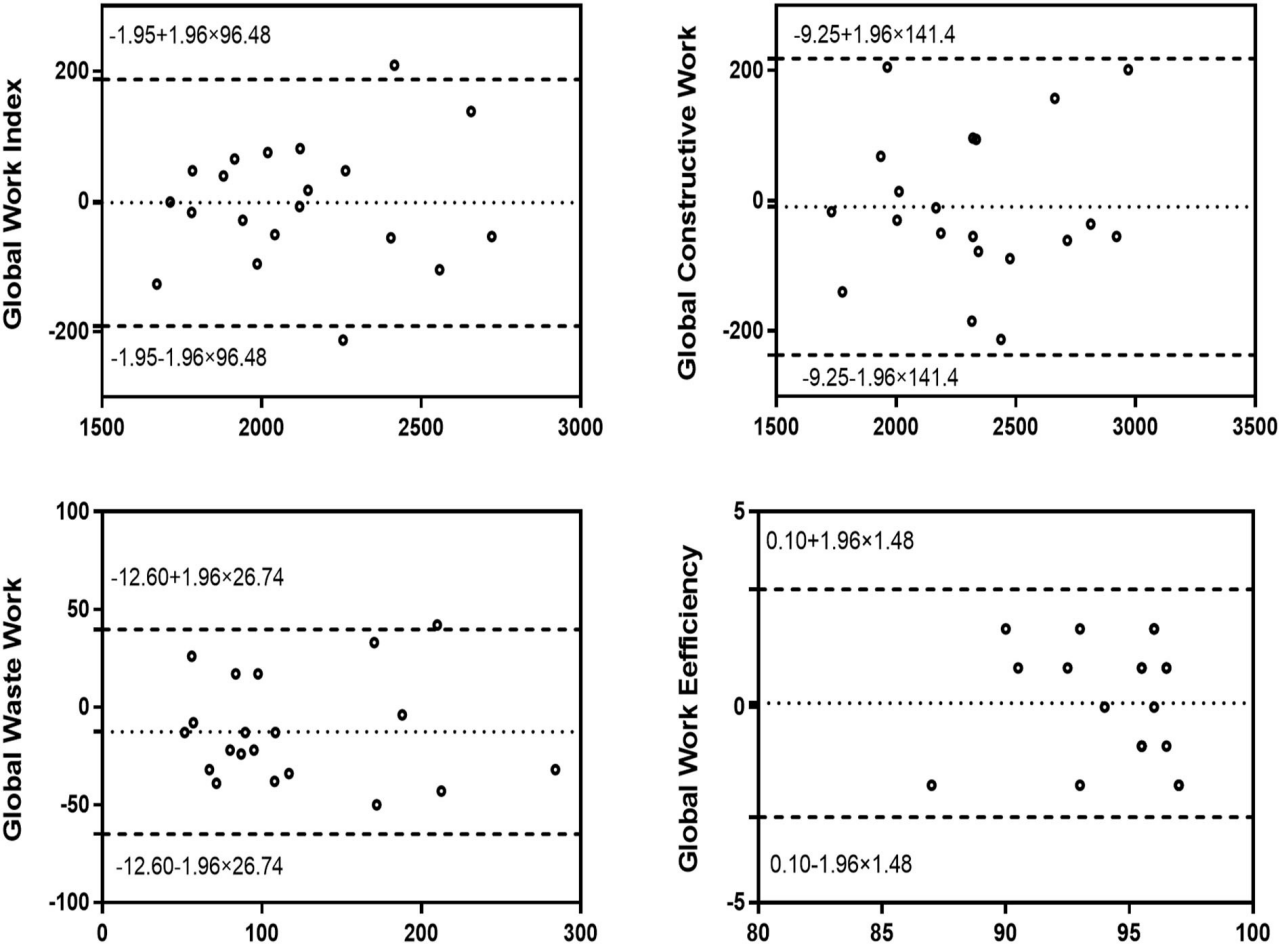
Bland–Altman analysis for assessment of the intra-observer variability of global work index, global constructive work, global wasted work, and global work efficiency. Dotted lines represent bias and 95% limits of agreement.

**Figure 5:**
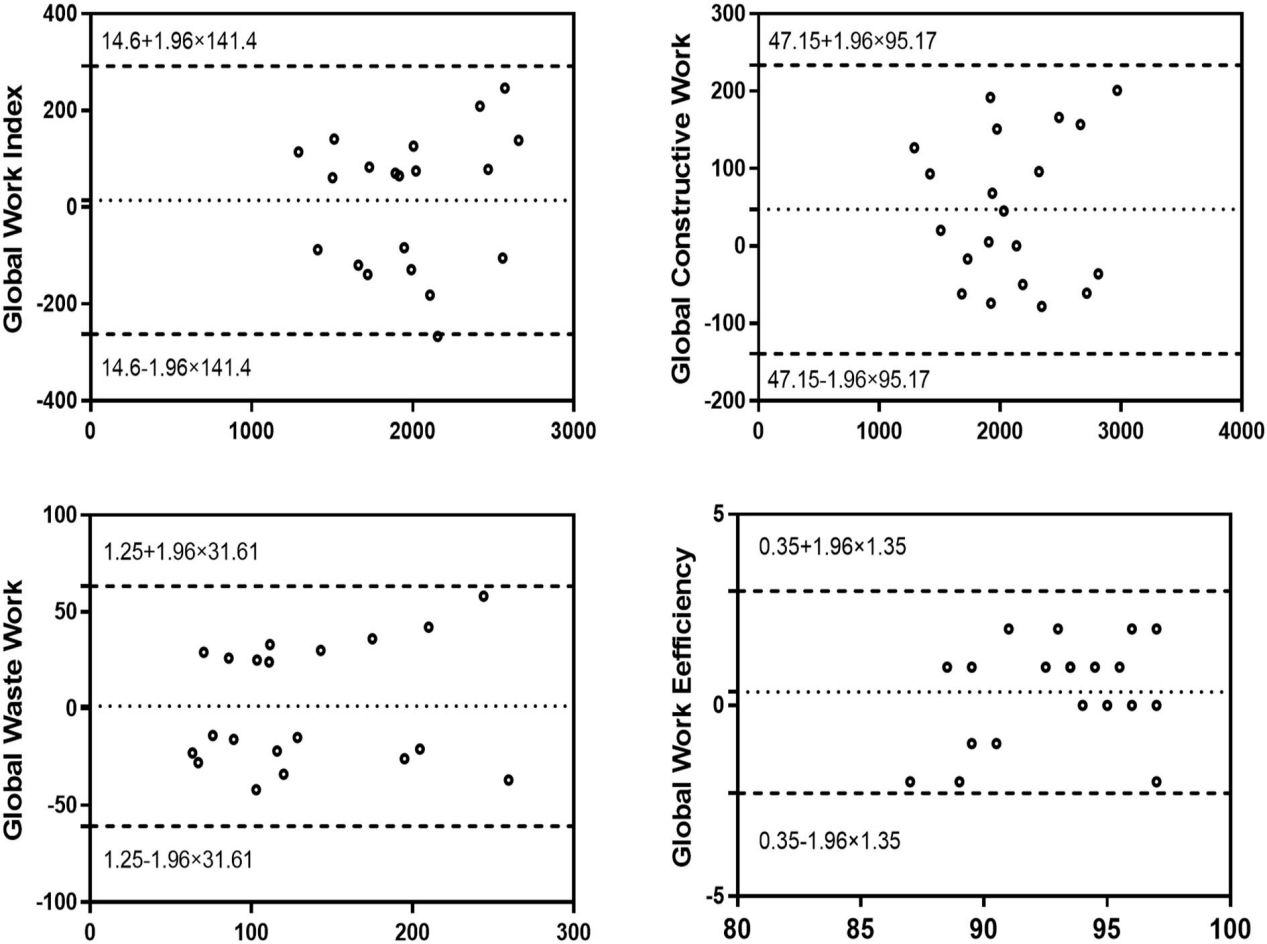
Bland–Altman analysis for assessment of the inter-observer variability of global work index, global constructive work, global wasted work, and global work efficiency. Dotted lines represent bias and 95% limits of agreement.

Figures 4, 5 show the intra-observer variability and inter-observer variability results obtained by the Bland-Altman analysis. The measured GWI, GCW, GWW, and GWE values were in high agreement.

## Discussion

Our study investigated that the global myocardial function of the left ventricle was significantly impaired in HCM patients with preserved LVEF, while GWI in patients with H-LVH was at a high level, and GWE was clearly decreased in the two groups. The diagnostic accuracy of IVST/LVPWT combined with GCW in differentiating HCM from H-LVH was 94.7%, thereby providing an incremental value for clinical diagnosis.

Although clinical and family history, along with physical examination, may narrow the differential diagnosis, the exact etiology can remain unclear. Cardiac imaging technologies are often needed to further differentiate. Echocardiography is the first choice for the diagnosis of left ventricular hypertrophy. Both HCM and H-LVH patients exhibited wall thickening, left atrial enlargement, diastolic dysfunction to different degrees, and normal LVEF. In addition, the location and degree of wall hypertrophy overlapped, and thus conventional echocardiography was of low value for diagnosing and distinguishing the two diseases (12, 13).

Traditionally, myocardial work assessment has been dependent on the left ventricular pressure-volume loop measured by a cardiac catheter, which can accurately evaluate the left ventricular systolic function in terms of myocardial work and metabolism, but it is invasive, limiting its feasibility in routine clinical practice (14). The NMWI is a new method for evaluating myocardium function (9). It obtained the left ventricular GLS using the echocardiographic speckle tracking technique, and the brachial artery cuff blood pressure was measured non-invasively instead of the pressure of the left ventricle, the deformation and afterload of the left ventricle were considered at the same time, thereby reflecting the myocardial function more objectively and accurately. Russell et al. (10) confirmed that the PSL areas obtained by the invasive or non-invasive measurement of the left ventricular pressure were highly consistent. Myocardial work assessment by NMWI is also correlated with the uptake of fluoro-deoxy-glucose at myocardial positron emission tomography scan which reflects myocardial metabolism. This new technology has been verified in many clinical diseases (15, 16), and some studies have already demonstrated that MW may be superior to GLS in assessing myocardial function (17, 18). Additionally, our study showed that NMWI parameters were significantly correlated with GLS and LVEF, and the repeatability was good, thereby further indicating the feasibility of NMWI for evaluating myocardial systolic function in H-LVH and HCM patients.

### Myocardial work in HCM and H-LVH

Although morphological features of HCM and H-LVH are similar in conventional echocardiography, their pathological mechanisms are quite different. HCM is an autosomal dominant genetic disease caused by mutations in the gene encoding sarcoprotein. H-LVH is a secondary pathological change, due to high peripheral vascular resistance and afterload, resulting in left ventricular compensatory hypertrophy.

Our study revealed that myocardial work in different hypertrophy types of disease was different. Compared to the control group, the absolute value of GLS, GWI, GCW, and GWE were significantly decreased in HCM patients, whereas the GWW was higher, indicating that the myocardial work of the global left ventricular was significantly damaged in HCM patients when LVEF was normal, This was also reported by few other authors (19). The damage to the myocardial work may be related to the hypertrophy of myocardial cells and the disordered myocardial fiber arrangement and interstitial fibrosis in HCM patients. In addition, uneven hypertrophy of the myocardium led to left ventricular remodeling and the unsynchronized contraction of each segment, thereby explaining the increased wasted work and decreased efficiency of the left ventricle in HCM patients (20).

A previous study showed that GLS could not reflect the true myocardial contractile function when the cardiac afterload changed (21). Its load dependency affects the diagnostic accuracy of myocardial function assessment as the increased afterload occurs concomitantly with decreased strain (22). In the present study, compared with the control group, the absolute value of GLS and GWE were lower in patients with H-LVH whereas GWI values were higher. Due to the long-term increase of systemic pressure and left ventricular wall compensatory thickening, the myocardial work correspondingly increased in patients with hypertension to resist the increased afterload and maintain a normal cardiac output (23). However, a long-term high afterload causes left ventricular remodeling, and the wall stress increases, resulting in impaired myocardial function (24). Our study's findings confirmed that NMWI does take into account the increased afterload and reflected the myocardial contractility of hypertensive patients in a more objective manner than GLS. Chan et al. (25) also showed that the GWI values were higher in hypertensive patients compared with those in the normal group, whereas GWE remained, which was not completely consistent with our results and it may be related to differences between the samples and groups.

### Discrimination of HCM and H-LVH

In our study, multivariate analysis showed that IVST/LVPWT and GCW were independent predictors for distinguishing HCM from H-LVH. The cut-off values for IVST/LVPWT and GCW when individually diagnosing HCM were 1.29 and 1,662 mm Hg%, respectively, with a sensitivity of 68.2 and 75.0%, specificity of 95.5 and 98.5%, and accuracy of 81.3 and 90.2%, respectively. However, the combination of IVST/LVPWT and GCW discriminated HCM and H-LVH with a higher predictive accuracy of 94.7%; the sensitivity was 90.0%, and the specificity was 100.0%. The clinical value of IVST/LVPWT in distinguishing patients with hypertension from HCM has been confirmed in previous studies (26, 27). As is known to all, abnormal ventricular wall hypertrophy is a typical feature of HCM patients, while symmetric (concentric) ventricular wall hypertrophy is the main manifestation of patients with hypertension. However, 13–31% of HCM patients present with symmetric septal hypertrophy, and 4–47% of hypertensive patients present with asymmetric septal hypertrophy, which limit the diagnosis accuracy (28). Our results suggested that the myocardial work combined with conventional echocardiography improves the diagnostic accuracy of the two diseases and provides incremental information for clinical differential diagnosis. In addition, within our group of research objects, GCW was weakly correlated with wall thickness (*r*=-0.40, *P*=0.000), which means that the wall thickness itself had relatively little influence on GCW. The suitability of GCW for use as a parameter to distinguish the two diseases may be attributed to its close correlation with the degree of myocardial fibrosis (19). A related pathological study showed that the ratio of myocardial fibrosis was significantly lower in H-LVH patients than in HCM patients (29). Additionally, Goncalves et al. (30) showed that GCW was the only parameter associated with left ventricular myocardial fibrosis > 20%.

### Limitations

This is a single-center cross-sectional study. Several limitations of the present study should be acknowledged. Firstly, the left ventricular pressure which was replaced by the cuff blood pressure for myocardial work analysis may affect the accurate evaluation of myocardial work. Secondly, echocardiographic evaluation of myocardial work might be influenced by the inadequate quality of ultrasound images. Thirdly, the regional myocardial work of 17 segments was not assessed. Fourth, different drugs therapy may have different influences on myocardial work parameters. In addition, most subjects of our study were young and middle-aged. Further studies with extended follow-up are needed to confirm our results and to validate the clinical value of myocardial work parameters, which will be undertaken in the next phase of the study.

## Conclusion

To summarize, global myocardial work has shown a significant difference between patients with HCM and those with H-LVH; NMWI offered new insights into the pathophysiology of both forms of hypertrophy. The combination of IVST/LVPWT with GCW may have additional clinical implications for the discrimination between HCM and H-LVH patients. Further prospective studies are thus needed to confirm our findings.

## Data availability statement

The original contributions presented in the study are included in the article/supplementary material, further inquiries can be directed to the corresponding author/s.

## Ethics statement

The studies involving human participants were reviewed and approved by the Ethics Committee of Fuwai Central China Cardiovascular Hospital. The patients/participants provided their written informed consent to participate in this study. Written informed consent was obtained from the individual(s) for the publication of any potentially identifiable images or data included in this article.

## Author contributions

QZ and CC collected data, analyzed it, wrote the manuscript, and reviewed and edited the manuscript. YLi, YLiu, DH, YW, YH, RL, and HZ performed this study. LL designed the study, reviewed it, and finally approved the submitted version. All authors read and approved the manuscript.

## Funding

This research was supported by National Natural Science Foundation of China (82071950, 81800287); National Natural Science Foundation of Henan Province for Excellent Young Scientists (202300410364); and Medical Science and Technology Research Project of Henan Province (SB201901099, LHGJ20190805, LHGJ20200084).

## Conflict of interest

The authors declare that the research was conducted in the absence of any commercial or financial relationships that could be construed as a potential conflict of interest.

## Publisher's note

All claims expressed in this article are solely those of the authors and do not necessarily represent those of their affiliated organizations, or those of the publisher, the editors and the reviewers. Any product that may be evaluated in this article, or claim that may be made by its manufacturer, is not guaranteed or endorsed by the publisher.
